# Cell Based Drug Delivery: *Micrococcus luteus* Loaded Neutrophils as Chlorhexidine Delivery Vehicles in a Mouse Model of Liver Abscesses in Cattle

**DOI:** 10.1371/journal.pone.0128144

**Published:** 2015-05-26

**Authors:** Sebastian O. Wendel, Sailesh Menon, Hamad Alshetaiwi, Tej B. Shrestha, Lauren Chlebanowski, Wei-Wen Hsu, Stefan H. Bossmann, Sanjeev Narayanan, Deryl L. Troyer

**Affiliations:** 1 Department of Chemical Engineering, Kansas State University, Manhattan, Kansas, United States of America; 2 Department of Diagnostic Medicine and Pathobiology, Kansas State University, Manhattan, Kansas, United States of America; 3 Department of Anatomy and Physiology, Kansas State University, Manhattan, Kansas, United States of America; 4 Department of Statistics, Kansas State University, Manhattan, Kansas, United States of America; 5 Department of Chemistry, Kansas State University, Manhattan, Kansas, United States of America; 6 University of Ha'il, Ha'il, Saudi Arabia; 7 Department of Chemistry, Augustana College, Rock Island, Illinois, United States of America; Second University of Naples, ITALY

## Abstract

The recent WHO report on antibiotic resistances shows a dramatic increase of microbial resistance against antibiotics. With only a few new antibiotics in the pipeline, a different drug delivery approach is urgently needed. We have obtained evidence demonstrating the effectiveness of a cell based drug delivery system that utilizes the innate immune system as targeting carrier for antibacterial drugs. In this study we show the efficient loading of neutrophil granulocytes with chlorhexidine and the complete killing of *E*. *coli* as well as *Fusobacterium necrophorum* in *in-vitro* studies. *Fusobacterium necrophorum* causes hepatic abscesses in cattle fed high grain diets. We also show in a mouse model that this delivery system targets infections of *F*. *necrophorum* in the liver and reduces the bacterial burden by an order of magnitude from approximately 2•10^6^ to 1•10^5^.

## Introduction

*Fusobacterium necrophorum* is a Gram-negative, rod shaped and obligate anaerobic bacterium, which is frequently associated with necrotic infections in animals [1]. *F*. *necrophorum* subspecies *necrophorum* is associated with liver abscess and foot rot in animals [2]. This organism is a major cause of infections including hepatic abscesses and necrotic laryngitis in feedlot cattle, both resulting from aggressive grain-feeding programs [3]. Foot rot and lameness in dairy and beef cattle and hepatic abscesses in feedlot cattle are of major economic concerns to the cattle industry [4], [5]. In feedlots, liver abscesses range from 12–32%, depending on various management and dietary factors [6]. In some feedlots, prevalence can be as high as 90–95% [7]. Cattle with liver abscesses have reduced feed intake, reduced weight gain, decreased feed efficiency, and decreased carcass dressing percentage. Liver abscesses and subsequently liver condemnations are of a major economic concern to beef producers and packers, as the liver accounts for roughly 2% of the weight of the carcass [3]. Economic loss incurred by the beef industry attributable to liver discounts alone has increased from $8.8 million in 1999 to $29.9 million in 2011 (NBQA audit 2011). Antibiotics such as tylosin used as feed additive causes significant reduction in the incidence of liver abscesses but does not prevent liver abscesses [8, 9].

The World Health Organization reports that there is a dramatic increase of microbial resistance against antibiotics [10]. As fewer antibiotics are discovered and made available for use, [11] a different drug delivery approach is urgently needed. There is increased concern among Public Health personnel and consumers over the use of antibiotics for growth promotion in food animals, and several European countries have banned the use of antibiotics as growth promoters (European Commission, 1998). The restriction of antimicrobial use will lead to a much higher incidence of liver abscesses and considerable economic loss to the cattle industry. Therefore, technologies and practices minimizing antimicrobial use are highly appealing.

Recently, liposomal delivery systems have been successfully employed in targeting single bacteria, as well as communities of biofilms [12]. Liposomal delivery systems are of increasing interest, because they are often capable of enhancing the therapeutic index of a drug while minimizing its adverse effects [13]. However, it is evident from the literature that the targeting efficacy of liposomes is limited to roughly 10 percent, because of their clearance from the bloodstream and the natural limitations of the available passive targeting mechanism. [14] Here, we evaluated the use of neutrophils as transport cells with an active mechanism to target the infection site [15].

Neutrophils (neutrophil granulocytes) are the most abundant white blood cell in most species of mammals, are an important part of the innate immune system and are known to be “first responders” to infections and environmental exposure [15]. Neutrophils are “experts” in recognizing and phagocytizing bacteria [16]; hence, if deactivated nonpathogenic bacteria could be loaded with antimicrobial drugs they should be readily taken up by neutrophils. We asked whether bacteria filled with broad-band antibacterial drugs could be transported within defensive cells to a site of infection and whether the therapeutic payload could be effectively released once the defensive cells have reached their target. Our choice of bacterium was *Micrococcus luteus*, because this bacterium is part of the natural human skin flora and has one of the smallest known genomes. [17]. Chlorhexidine was selected as broad-band antimicrobial because it is a bactericidal and bacteriostatic as well as a topical disinfectant [18]. It is commonly used in mouthwash and contact lens cleaning solutions. Chlorhexidine’s mechanism of action is the attachment to the bacterial cell wall and membrane due to its positive charge [19]. It then disrupts the integrity of the semipermeable membrane, allowing an uncontrolled flow of ions that kills the cell [20, 21]. The uptake time for CHX has been reported as very rapid at about 20 seconds [22]. Although this study is focused on an animal pathogen using a mouse model, the concept established in the study will also be applicable to human diseases.

## Materials and Methods

### Ethics Statement

This study was carried out in strict accordance with the recommendations in the Guide for the Care and Use of Laboratory Animals of the National Institutes of Health. The protocol was approved by the Institutional Animal Care and Use Committee at Kansas State University (IACUC approved protocol #3298).

### Loading of *M*. *luteus* with CHX

*M*. *luteus* (ML) was grown in an overnight culture in LB broth at 37°C and 200 rpm. The cells were separated from the medium and washed with PBS buffer. To load *M*. *luteus* with chlorhexidine (CHX), the cell pellet of 1 ml cell-suspension was resuspended in concentrated CHX (the stock solution is 2% w/v). The use of PBS buffer or sodium chloride solution to dilute the CHX solution led to unwanted precipitations.

The amount of CHX per 1·10^8^ cells of *M*. *luteus* was quantified using HPLC. CHX-loaded *M*. *luteus* cells were lysed with a 1% Triton X Tris lysis buffer (pH 7.2). The cell fragments were removed by centrifugation and the supernatant was analyzed via HPLC (Column: Phenomenex Kinetex 5u XB-C18, Detector: Waters 2998 Photodiode Array Detector). A 20 μl sample of the supernatant was injected for analysis and the UV-Vis spectrum was recorded from 400 to 700 nm. The absorption peak of CHX occurring at 630 nm was used for calibration and data analysis.

### Thioglycollate Induced Peritonitis for isolation and *ex-vivo* use of neutrophils

Syngeneic mice were used to isolate circulating leukocytes for loading with appropriate antimicrobials. Mice were injected with 2.5 ml of autoclaved, aged thioglycollate medium (Fisher Scientific) intraperitonially. Twelve hours later, mice were placed under isoflurane anesthesia (2.0%-4.0%) and neutrophils were isolated by peritoneal lavage. The mice were then euthanized. All studies were approved by Institutional Animal Care and Use Committee at Kansas State University.

### Flow Cytometry Analysis of Neutrophils

After the extraction of neutrophilsfrom the host mouse, cells were counted and resuspended in RPMI medium at a concentration of 5•10^5^ cells/ml (cut-off for the flow cytometer). The cells were subsequently exposed to dead bacterial samples ranging from 10 to 100 bacterial cells per neutrophil granulocyte. The cell cultures were incubated for two hours at 37°C in a 25ml T-flask. Then the protocol for the PI/annexin 5 apoptosis assay was carried out [23].

### Zeta Potential Measurements

Zeta potential measurements were performed using the ZetaPALS Zeta Potential Analyzer (Brookhaven Instruments Corporation), as described previously [24]. *M*. *luteus* was suspended in 1 X PBS buffer at 1•10^9^ cells/ml. The influence of CHX-hydrochloride on the zeta potential was studied over a range of concentrations from 0μg/ml to 1000μg/ml CHX.

### *In-vitro* drug delivery against *E*. *coli* and *F*. *necrophorum*

The extracted neutrophils (PMN) were loaded with 20 bacteria per neutrophil (4–6·10^6^ neutrophils per sample). The samples were incubated at 37°C for two hours. Sample types were PMN + ML, PMN + ML modified with CHX, PMN and RPMI buffer.

A starter culture of *E*. *coli* C600N was grown overnight from a single colony in LB broth containing ampicillin at a final concentration of 50ng/ml and nalidixic acid at a final concentration of 12ng/ml. A 0.1ml volume of the overnight culture was added to 5ml of fresh LB broth containing no antibiotics, and grown to an O.D._600_ of 0.3. Following centrifugation the bacterial pellet was resuspended in 1X PBS to achieve 1·10^7^ CFU/ml. The bacterial culture was then incubated with 10% v/v complement inactivated mouse serum for 20 minutes at 37°C to opsonize the bacteria. The opsonized bacteria were then mixed with equal amount of PMNs (1:1 MOI and 1:1 volume of each) and incubated at 37°C for 90 minutes, on a rocker platform. The tubes were then spun down at 5,000 RPM for 5 minutes, the supernatant was discarded and the pellet was resuspended in 100μl of 1X PBS. 100μl of 1% Triton/Tris was added to the resuspended pellet to lyse the eukaryotic cells, and was gently mixed to ensure that no bubbles were formed. This mixture was allowed to sit at room temperature for 5 minutes. 800μl of LB broth was added to the mixture to bring up the final volume to 1ml, which was then serially diluted and plated on LB agar plates containing a final concentration of 50ng/ml ampicillin and 12ng/ml nalidixic acid to enumerate the bacteria.

A starter culture of *F*. *necrophorum* subsp. *necrophorum* strain 8L1 was grown overnight from a single colony in pre reduced anaerobically sterilized brain heart infusion broth (PRAS-BHI). A 0.3 ml aliquot of the overnight culture was added to 10ml of fresh PRAS-BHI broth and grown to an O.D._600_ of 0.7. The bacteria were spun down to form pellets. Resuspension of bacterial pellet in PBS, opsonization, incubation with neutrophils, lysis of eukaryotic cells from the suspension, serial-dilution were all performed as described above for *E*. *coli*, but under strict anaerobic conditions. Enumeration was performed by plating on blood agar followed by incubation at 39°C in an anaerobic chamber (Forma Anaerobic System; Thermo Scientific).

### Preliminary toxicity Study

In order to determine whether chlorhexidine gluconate was toxic to mice, we conducted *in-vivo* toxicity assays. Briefly, ten week old BALB/c mice were given tail vein intra-venous (IV) injections containing approximately one million neutrophils loaded with an average of 20 cells of *M*. *luteus* modified with either 1% or 2% chlorhexidine gluconate (CHX). The animals were observed for 5 days post challenge to determine whether they demonstrated any clinical signs of toxicity. At the end of 5 days, the mice were euthanized and their kidneys, liver, brain, lung, spleen and heart were fixed in 10% formalin for histopathological evaluation.

### Mouse-treatment Study

*Fusobacterium necrophorum* subsp. *necrophorum* strain 8L1 was grown overnight from a single colony in PRAS-BHI. 0.3ml of the starter culture was added to 10ml of fresh PRAS-BHI broth and grown to an O.D._600_ of 0.7. 1 ml of this culture was diluted 1:40 to achieve a final concentration of approximately 4•10^6^ CFU/ml [25]. 400μl of the diluted bacteria was injected intraperitonially into 10 week old BALB/c mice. The mice were observed for two days before they were treated with neutrophils carrying antimicrobial cargo or controls.

10 week old BALB/c mice were randomly assigned into 6 groups with 9 mice per group. On day zero, all mice were infected intraperitonially with an infectious dose of *F*. *necrophorum*. On day 3 of the experiment, all mice were given intravenous tail vein injections containing 100μl of the following treatments or controls- mice in group 1 were treated with one million neutrophils containing an average of 20 cells of *M*. *luteus* loaded with chlorhexidine. Mice in group 2 were treated with one million neutrophils containing unmodified, heat deactivated *M*. *luteus*, mice in group 3 were treated with one million neutrophils and mice in group 4 were treated with 1X PBS. The animals were monitored for clinical signs for 5 days after treatment and were euthanized if any clinical signs developed. The mice were euthanized 5 days post treatment and all mice were examined post mortem for abscesses in the livers. Livers of the mice were weighed and homogenized in a tissue homogenizer for 1 min in modified lactate (ML) broth [26]. The *F*. *necrophorum* bacterial load in the homogenate was enumerated using most probable number (MPN) analysis [27] using ML broth. Homogenized liver tissue samples were streaked on blood agar plates for bacterial isolation and identification using Rapid ANAII system (Thermo Scientific). Liver, lung and spleen were fixed in formalin for histopathological evaluation.

## Results

### Bacterial Loading and CHX retention

CHX was identified by its absorption peak at 630 nm via HPLC. The concentration of CHX in the bacterial sample was determined by a calibration curve with the range 500–4000 μg CHX/ml in 500 μg increments. The calculated concentration of CHX is 204.6± 19.6 μg CHX/10^8^ cells of *M*. *luteus*.

The uptake of CHX loading also was observed over a 4 hour time frame in one hour increments showing that with an increased loading time more CHX is taken up. The results, however, are not significant (p>0.05) and all samples for *in-vitro* and *in-vivo* experiments have been loaded for 24h to ensure a steady state. Genuit et al., reported that uptake of chlorhexidine by bacteria occurs within 30 seconds [28]. This explains why the uptake of chlorhexidine did not increase significantly between the first and the fourth hour.

**Fig 1:**
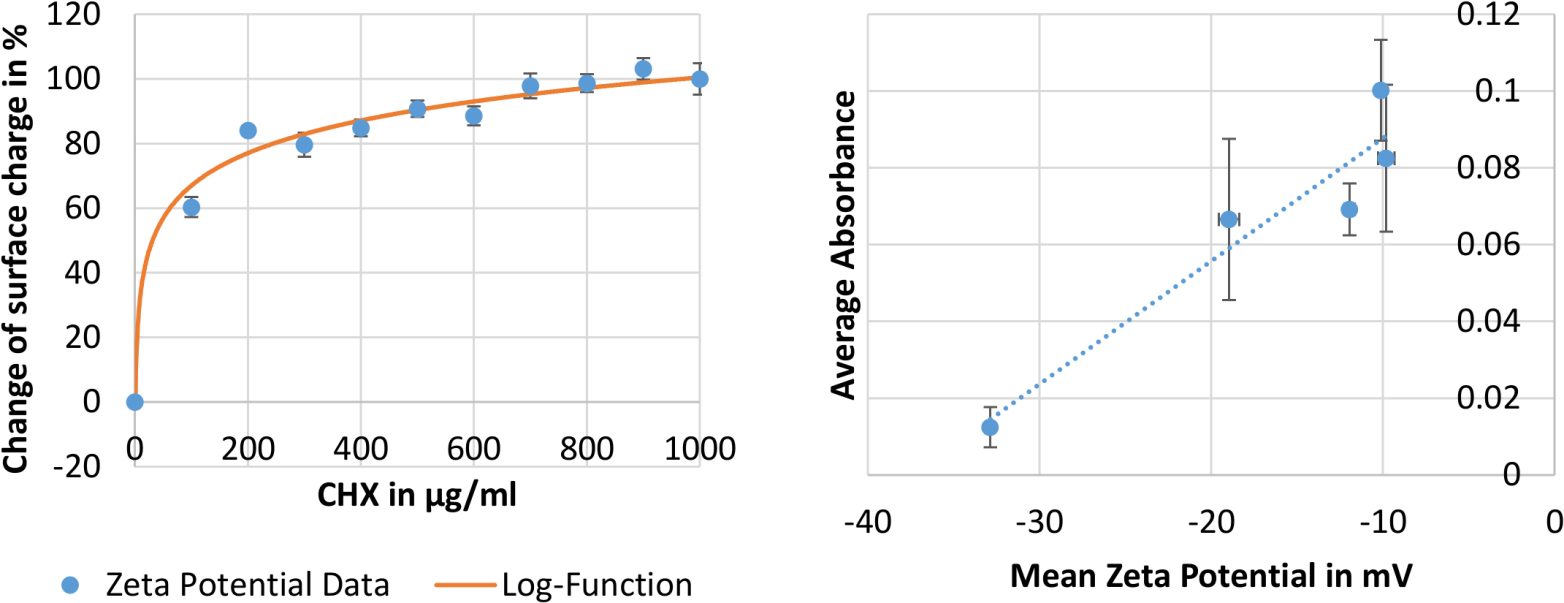
Zeta potential data (surface charge) for *M*. *luteus* loaded with CHX-hydrochloride over a range of concentrations from 0 μg/ml to 1000 μg/ml CHX. The data has been normalized (0%-100% equals an increase of the zeta potential from -32.86 ± 1.88 mV to -9.63 ± 2.22 mV) in order to fit a logarithmic function (%ζ = 33.48•log(CHX-concentration)). The panel on the right shows correlation of the measured zeta potential (x-axis) with the measured absorption of CHX in the lysate (y-axis). A linear trend with an R^2^ value of 0.9045 is also shown.

Another method to assess CHX loading and, more importantly, where in the bacterial cell CHX is located, is a correlation of zeta potential measurement and UV-Vis analysis of loaded CHX. Since the mechanism of action for CHX is governed by the attraction of the positively charged CHX molecule to the negative surface charge of the bacterial cell an increase of this surface charge can be measured. Fig 1 (left panel) shows the increased zeta potential for a CHX-hydrochloride concentration from 0 μg/ml to 1000 μg/ml loading stock solution in 100μg/ml increments. The data has been normalized to allow a nonlinear regression of the underlying logarithmic shape of this dataset. The zeta potential reaches from -32.86 ± 1.88 mV (0%) at 0μg/ml CHX to -9.63 ± 2.22 mV (100%) at 1mg/ml CHX.

We subsequently correlated the zeta potential data to the actual amount of CHX loaded into the bacterial cell as we detect it by lysing the cells and measuring the CHX concentration in the lysate. The detected linear correlation in Fig 1 (right panel) obtained by the least squares regression model, suggests that most of the CHX is bound on the surface of the cells or integrated into the cell wall rather than taken up into the cell’s cytoplasm. If CHX would enter the cytoplasm a further increase of CHX concentration in the cell lysate would be expected, while the zeta potential is maintained at its upper limit of roughly -10mV. However this is not the case. A possible explanation is that the negative surface charge has reached a level too weak to further attract the positively charged CHX molecules.

The ability of *M*. *luteus* to retain CHX it has taken up is vital to ensure that the neutrophil’s quality as a drug carrier is not impaired by cytotoxic effects of released CHX on the way to the site of the infection.

Data (not shown) indicates that after 5 days, 5% of the loaded CHX are released by the cells into the buffer. Given the average lifespan of neutrophils of 3–5 [29] days this presents a satisfying result in terms of protecting the neutrophil carrier cell from cytotoxic CHX effects.

### Neutrophil loading and survival *ex vivo*

**Fig 2:**
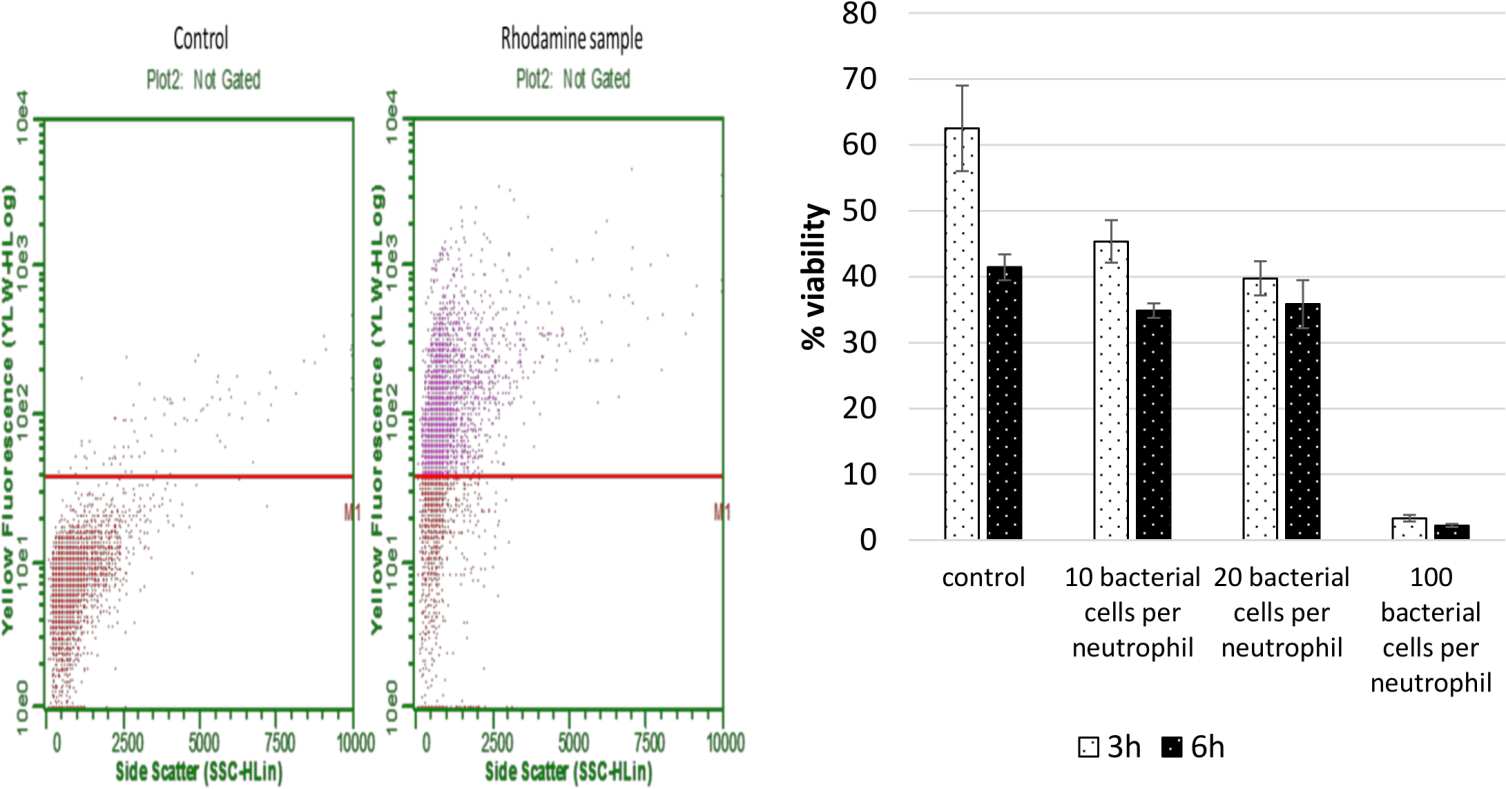
Comparison of the fluorescence of an unloaded neutrophil population with a neutrophil population loaded with rhodamine-modified *M*. *luteus*. Panel on the right shows neutrophil survival after 3 and 6 hours for ratios of 10, 20 and 100 bacteria/neutrophil determined by PI/annexin 5 apoptosis assay and flow cytometry.

To determine the *ex-vivo* neutrophil loading efficiency for modified bacteria, *M*. *luteus* was loaded with a fluorescent rhodamine-B dye. The neutrophil sample was analyzed via flow cytometry and compared to an unloaded neutrophil sample. Fig 2 (left panel) shows that approximately 70% of the neutrophil population had taken up the rhodamine-b modified *M*. *luteus* after a 2h loading period.

*Ex-vivo* neutrophil survival during the loading phase is critical for this drug delivery approach. Neutrophils do not survive for more than a day *ex-vivo* and additional stress by exposure to a bacterial sample can further reduce the survival time dramatically. It has been shown that phagocytic activity increases the oxygen consumption of neutrophils by the factor of two [30]. Fig 2 (right panel) shows that ratios of 10–20 bacteria per neutrophil give reasonable survivability of the neutrophil when compared to the control group. If exposed to a ratio of 100 bacteria per neutrophil the survival drops below 10% for both time points.

### *In-vitro* Results

**Fig 3:**
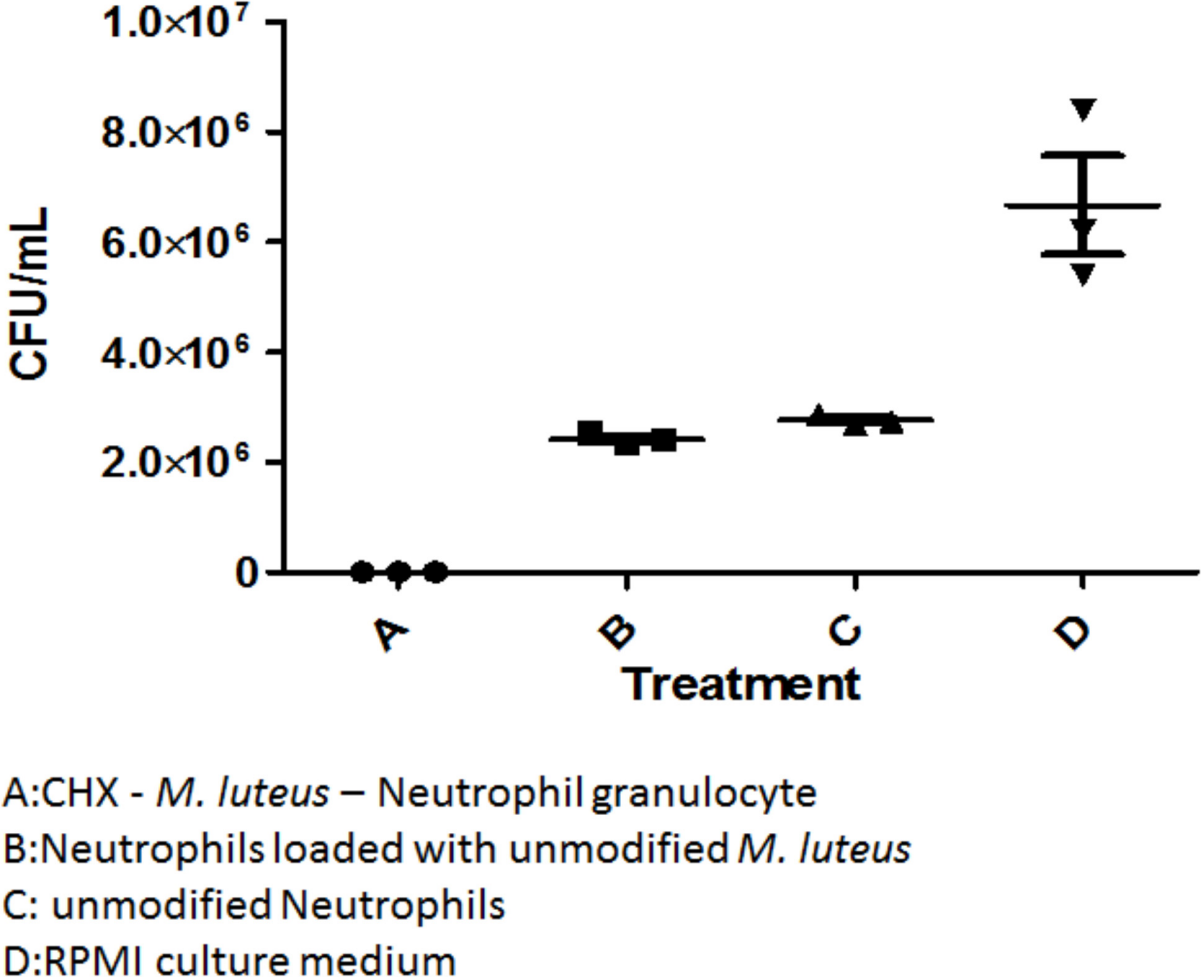
*In-vitro* tests of the cell based CHX drug delivery system on *E*. *coli* C600N. Here *E*. *coli* C600N was exposed to the following test groups: A is the CHX—*M*. *luteus*—neutrophil delivery system, B is the first control group with neutrophils loaded with unmodified *M*. *luteus*, C is the second control group with just unmodified neutrophils and D is the third control group with plain RPMI culture medium.

**Fig 4:**
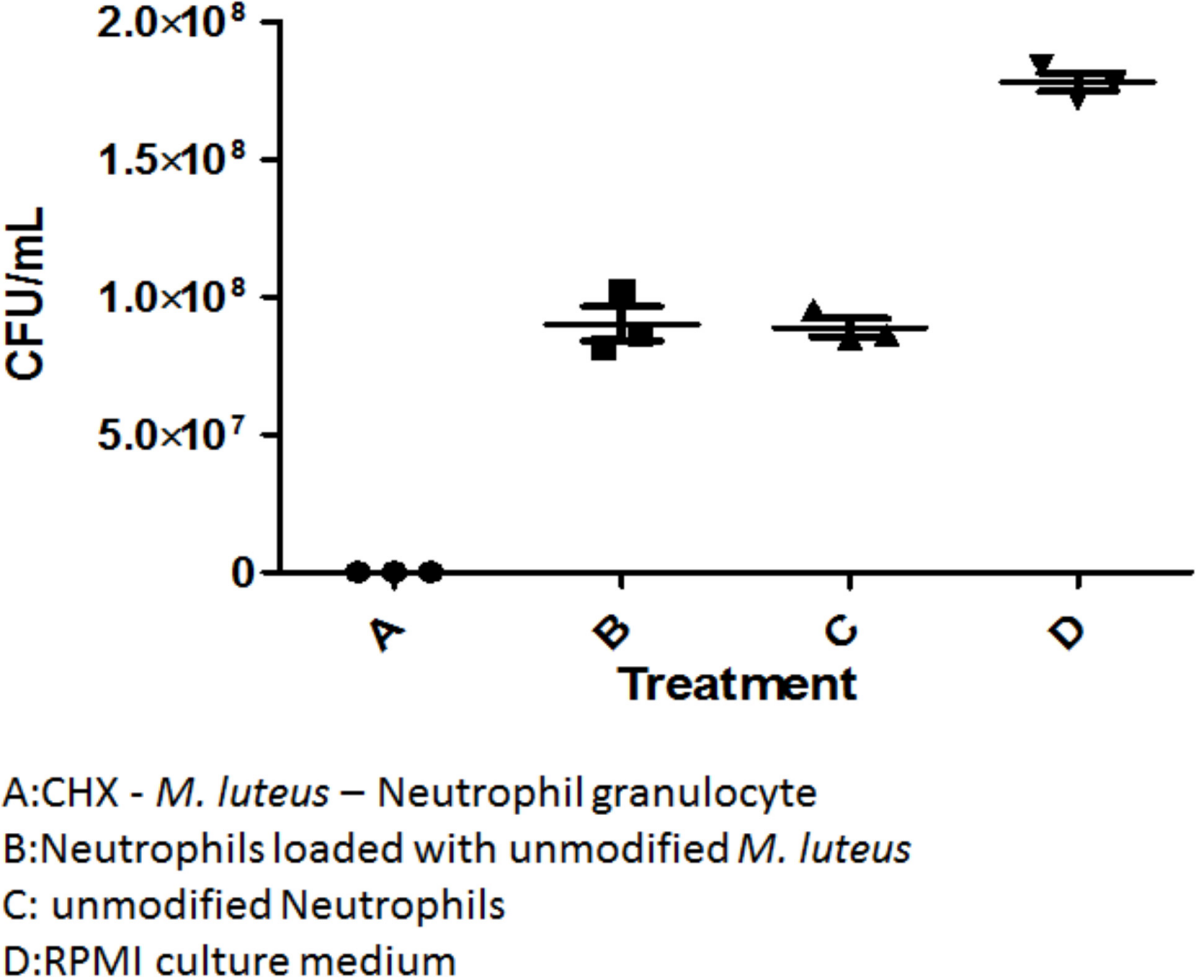
*In-vitro* tests of the cell based CHX drug delivery system on *Fusobacterium necrophorum*. The bacterium was exposed to the following test groups: A is the CHX—*M*. *luteus*—neutrophil delivery system, B is the first control group with neutrophils loaded with unmodified *M*. *luteus*, C is the second control group with just unmodified neutrophils and D is the third control group with plain RPMI culture medium.

The delivery system was tested *in-vitro* on *E*. *coli* and *F*. *necrophorum* cultures. In the literature, the MIC-value of CHX is 0.25–8 μg/ml [28]. In our assays, the MIC_50_ value of CHX for *E*. *coli* C600N was 19.5 ug/ml, and the MIC_50_ value of CHX for *F*. *necrophorum* strain 8L1 was 9 ug/ml. These values were exceeded with a CHX concentration of 60.4 ± 4.7 μg/ml in the *in-vitro* tests. In both cases, the targeted bacterium was killed completely in the treatment group. Control groups containing neutrophils or neutrophils loaded with unmodified *M*. *luteus* reduced the bacterial count when compared to the no-neutrophil-control group, but failed to completely eliminate the bacteria, leaving viable CFUs in the range of 10^6^–10^8^. This drop in the CFU count can be attributed to the increased release of bactericidal reactive oxygen species from neutrophils as shown by [31]. Fig 3 shows the results of the *in-vitro* tests using the neutrophil based CHX delivery system on *E*. *coli* C600N. The CHX treatment group was able to kill all *E*. *coli* C600N so that no colonies were found on LB agar plates. Similarly, Fig 4 shows the complete killing of *F*. *necrophorum* using the neutrophil based CHX delivery system. Again the control groups containing live neutrophils show a reduced CFU count that can likely be attributed to the bactericidal properties of the phagocyte.

### *In-vivo* Results: Toxicity and safety assessment

A preliminary toxicity study showed no toxic effects on the mice, and all of the mice appeared normal and healthy after treatment with neutrophils loaded with 1% or 2% chlorhexidine at any time during the five day observation period after transplant. Moreover, microscopic examination of tissue sections by a pathologist (S.N.) revealed no lesions.

**Fig 5:**
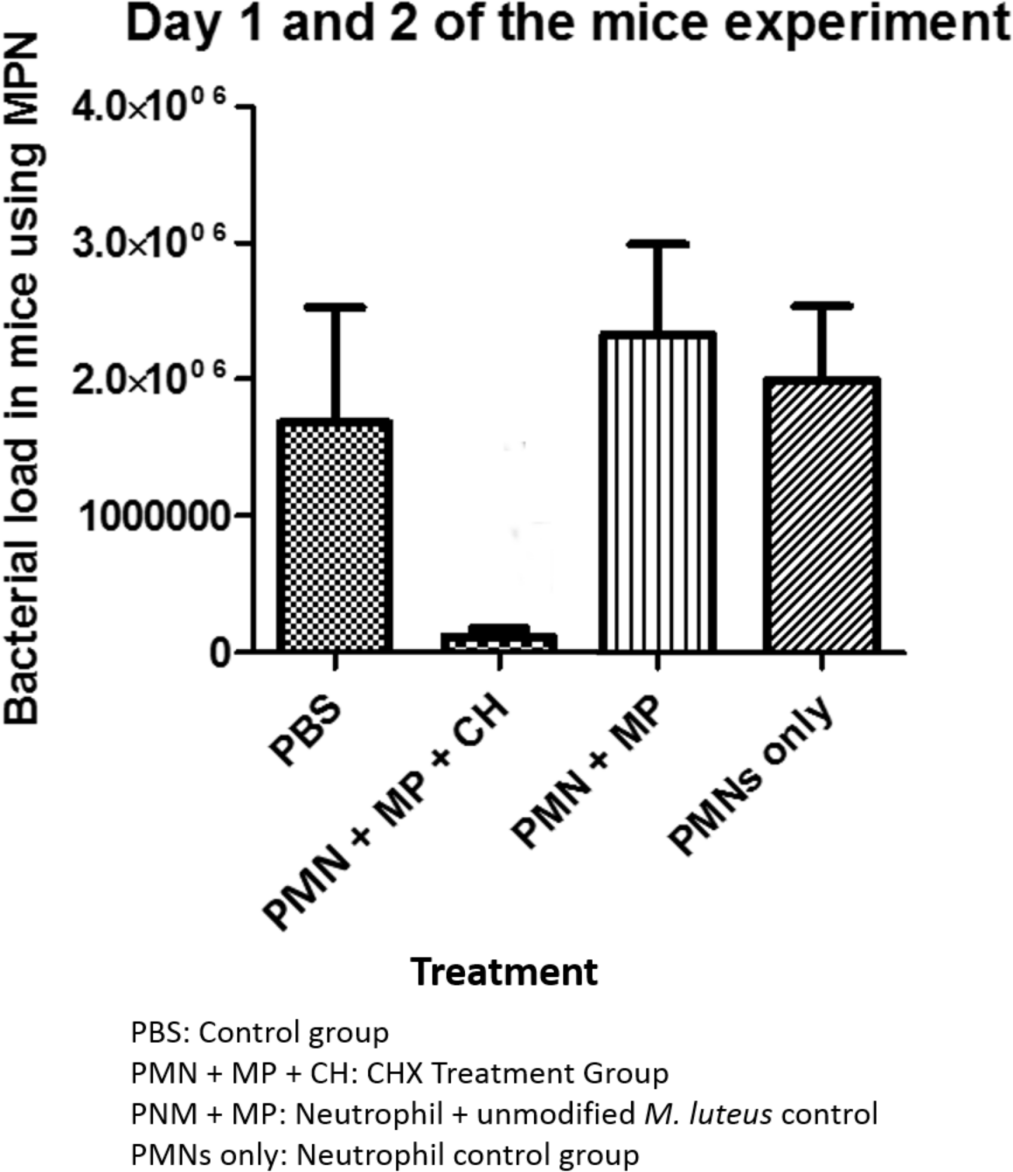
Results of the *in-vivo* experiment. MP + CHX is the treatment group containing neutrophils loaded with CHX modified *M*. *luteus*. The PBS group is the control group containing pure RPMI medium while PMN and PMN + MP represent the neutrophil control groups without and with unmodified *M*. *luteus*.

In the final *in-vivo* mouse experiment the CHX treatment group showed significant results (p<0.05, Pairwise Two-Sided Multiple Comparison Analysis; Dwass, Steel, Critchlow-Fligner Method [32, 33, 34]) against all control groups. Each mouse in the CHX treatment group received a dose of 0.056 mg CHX carried in the loaded neutrophils. The CFU count was reduced by an order of magnitude from an average of approximately 2·10^6^ in the control groups to 1·10^5^. Fig 5 shows the CFU count results of homogenized and plated liver samples.

**Fig 6:**
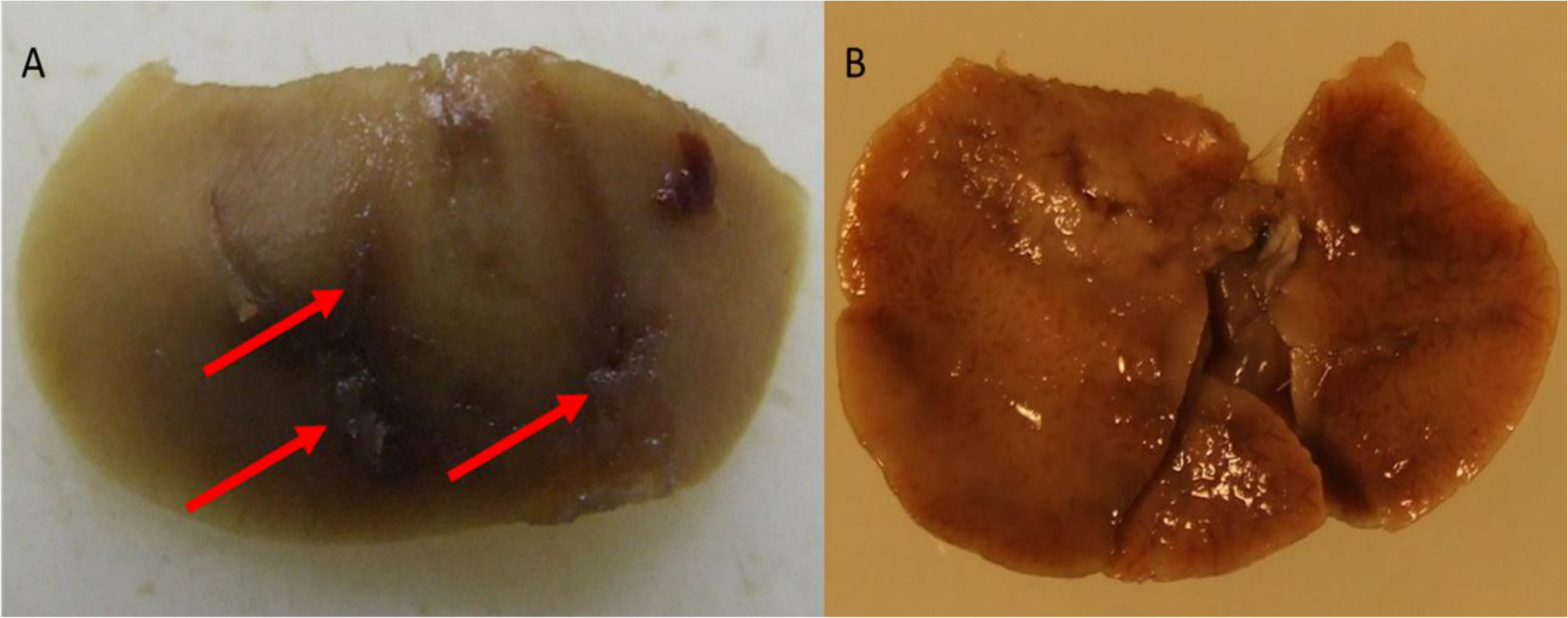
Image A shows a mouse liver from the control group with lesions (marked by the arrows) caused by *Fusobacterium necrophorum*. Image B shows a liver from the CHX treatment group with no visible lesions.

A subsequent spectroscopic analysis of the mouse liver samples showed no traces of residual CHX five days after the treatment injection. The damage caused to an untreated liver by *F*. *necrophorum* can be seen in Fig 6, while livers of the treatment group showed no lesions.

## Discussion

Here, we present the following findings: (1). A non-pathogenic bacterium (*Micrococcus luteus*) can be used as a vector for efficiently loading neutrophils with drugs *ex-vivo*, (2). Chlorhexidine (a broad spectrum disinfectant) can be efficiently taken up and trapped by the bacteria, (3). Mouse neutrophils containing the therapeutic cargo can kill *F*. *necrophorum in-vitro*, and (4) Mouse neutrophils loaded *ex-vivo* with the *M*. *luteus* containing antimicrobial drugs can be used as vehicles for targeted therapy of liver abscesses in mice caused by *F*. *necrophorum*.

Thus, the bacterium *M*. *luteus* is used as a liposome-like protective shell for the active ingredient, chlorhexidine. The additional stability provided by the bacterial cell wall is key to the successful transport of CHX. In our experience liposomes are disrupted by CHX (data not shown). While liposomes rely on their size (passive targeting) and possible targeting ligands on their outer surface to deliver the drug to its target, the cell based approach described here is characterized by a much more specific targeting mechanism as well as an active form of movement. Neutrophils, the targeting component of this drug delivery system, are recruited via chemotaxis into infected tissue [35]. Bacterial antigens naturally enhance the uptake of modified, drug carrying *M*. *luteus* by neutrophils while the structure of cell wall and membrane represent a natural version of a caged liposome with superior stability against shear- and osmotic stresses. This is a major difference to the bacterial ghost system where inner and outer membranes of Gram-negative bacteria fuse to create an empty bacterial shell. While this has the advantage of a minimal introduction of bacterial DNA into the host organism the bacterial ghost shell releases 40% of the drug it carries within one day. Using intact *M*. *luteus* as drug carrier has the advantage that approximately 95% of the chlorhexidine is retained within the dead bacterial cell during transport after uptake by the neutrophil. On the other hand, apoptosis of the neutrophil, which naturally occurs after reaching the target, is a dramatic event, which accomplishes the release of a therapeutically effective amount of chlorhexidine. Importantly, the *M*. *luteus* protects the neutrophil granulocyte, our targeting carrier, from cytotoxic effects of the drug and enhances uptake via phagocytosis. If liposomes were used, they would fuse to the neutrophil and release the free drug into the interior, where it could be cytotoxic.

Chlorhexidine is a potentially suitable bactericidal drug showing activity even against MRSA strains [36, 37]. Using neutrophils as delivery vehicle has the advantage of a rapid targeted delivery to an infection site due to a fast inflammation response. This allows localized treatment with low drug dosages. The short lifespan of neutrophils [28] guarantees a quick release of the drug.

This cell-delivered antibacterial therapy was efficacious in the mouse model even though only a small amount of chlorhexidine was administered (60 micrograms in 1.3·10^6^ neutrophils), and only one dose was administered. Multiple daily injections of the cell therapy may have completely eliminated all bacteria. It is also possible that the cell-based therapy discussed here could be further enhanced by combining two or more agents in the bacteria. For example, silver has been shown to greatly enhance the efficacy of antibiotics, even in cases of drug resistance [38].

Effective antibacterial drugs exist, even for multidrug resistant bacteria, but they are limited by severe toxicity issues. For example, colistin, a polymyxin antibiotic discovered more than fifty years ago but discontinued due to its toxicity is now again in the clinic to treat cystic fibrosis patients, for which there is no other recourse [39]. The approach presented here could allow such drugs to be used more effectively because (1) cells are used as a Trojan horse to shield normal tissues and cells from the drug, and (2) only very small amounts of the drug are required.

Bacterial resistance to antimicrobials is now a global threat to humanity. The findings presented here may offer the prospect of a new armament in the perpetual arms race against multidrug resistant bacteria, since it utilizes a cell-based targeted approach for single or combinatorial delivery of antiseptics or antibiotics that would otherwise be toxic. It is intriguing that a very small amount of drug only administered once can be leveraged into a therapeutic benefit when actively targeted to the site of infection by neutrophils.

## Supporting Information

S1 File HPLC raw data of CHX-loading quantification. (XLSX)

S2 File Raw data of anti *E*. *coli* and anti *F*. *necrophorum in vitro* experiments. (XLSX)

S3 File *In vivo* data of mice MPN liver scores. (XLSX)

S4 File Loaded neutrophil survival data. (XLSX)

S5 File *M*. *luteus* zeta potential and correlation to amount loaded. (XLSX)

S6 File CHX retention by *M*. *luteus* day 1–5. (XLSX)

S7 File Direct observation of CHX in *M*. *luteus*. (XLSX)

S8 File Quantification of the loaded neutrophil population. (XLSX)
